# Adenoma with complete circumferential involvement of the ileum and ileocecal valve successfully removed by traction-assisted endoscopic submucosal dissection

**DOI:** 10.1055/a-2513-2734

**Published:** 2025-02-06

**Authors:** Simona Agazzi, Eukene Rojo, Clara Yzet, Jérôme Rivory, Louis Jean Masgnaux, Elena De Cristofaro, Mathieu Pioche

**Affiliations:** 1 18631 Gastroenterology and Digestive Endoscopy, Fondazione IRCCS Policlinico San Matteo, Pavia, Italy; 2 16517 Gastroenterology Unit, Hospital Universitario de la Princesa, Madrid, Spain; 3 36673 Gastroenterology, Amiens-Picardy University Hospital, Amiens, France; 4 36609 Gastroenterology and Endoscopy Unit, Hopital Edouard Herriot, Lyon, France; 5 60259 Gastroenterology, Department of Systems Medicine, University of Rome Tor Vergata Faculty of Medicine and Surgery, Rome, Italy

The ileocecal valve (ICV) is one of the most difficult locations for endoscopic submucosal dissection (ESD) because of limited scope maneuverability, the presence of fatty tissue, and particular anatomic features 1[1] . Consequently, the en bloc and R0 resection rates are reported to be lower than for other locations 2[2] 3[3] . Lesions covering ≥75% of the ICV, involvement of the anal lip or involvement of more than two sites on the ICV are reported to be risk factors for poor outcomes 4[4] . Traction-assisted ESD has recently emerged as a technique that facilitates ESD, improving the rates of R0 resections in this challenging location 4[4] 5[5] .

We report a case of a 69-year-old patient who presented with normocytic anemia and rectal bleeding. A colonoscopy was performed showing a 6-cm granular laterally spreading tumor with macronodules on the cecum (0-IIa+Is, Paris classification). The lesion extended over the ICV, involving 100% of the circumference, and involving the distal ileum. The lesion was suggestive of adenomatous histology, Kudo 4, Sano 2, and JNET 2a after evaluation with white light and narrow-band imaging.

An ESD was performed that took 90 minutes, using an adaptable multitraction device (A-TRACT 2+2) ( Fig. 1Abb. 1 , Video 1Video 1 ). First, a complete incision of the ileal margin was performed, followed by a complete incision on the colonic side. Traction was then placed at four points, which allowed opening of the ICV and improved the exposure of the edge of the lesion ( Fig. 2Abb. 2 ). Tightening of the device allowed increased traction of the lesion outside the ileum.

**Fig. 1:**
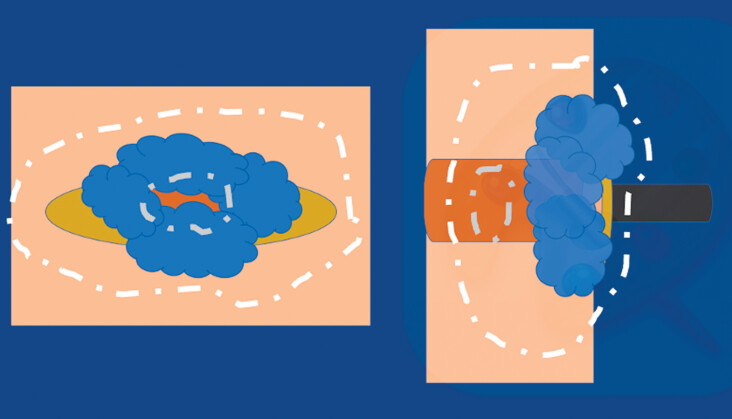
Removal of lesion with circumferential involvement of the ileocecal valve by a “doughnut” endoscopic submucosal dissection (ESD) assisted by an adaptable traction system.

**Fig. 2:**
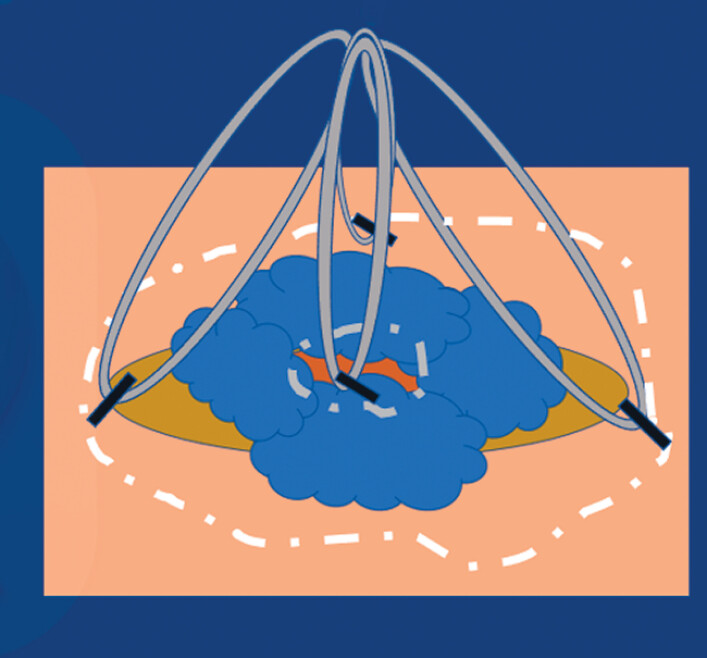
Placement of the A-TRACT traction system.

We resected the lesion en bloc without immediate complications. The defect was partially closed with two clips, to allow the opening of the valve. The patient developed delayed bleeding which required endoscopic thermal hemostasis and a few additional days of monitoring.

The histology report showed an R0 resection of a high grade dysplastic adenoma.

Endoscopic submucosal dissection (ESD) of a neoplastic adenoma with complete circumferential involvement of the ileum and ileocecal valve, by a “doughnut” resection facilitated by an adaptable multitraction device.Video 1

Endoscopy_UCTN_Code_TTT_1AQ_2AD_3AD

## Notes

Endoscopy E-Videos https://eref.thieme.de/e-videos
*E-Videos* is an open access online section of the journal *Endoscopy* , reporting on interesting cases and new techniques in gastroenterological endoscopy. All papers include a high-quality video and are published with a Creative Commons CC-BY license. Endoscopy E-Videos qualify for HINARI discounts and waivers and eligibility is automatically checked during the submission process. We grant 100% waivers to articles whose corresponding authors are based in Group A countries and 50% waivers to those who are based in Group B countries as classified by Research4Life (see: https://www.research4life.org/access/eligibility/ ). This section has its own submission website at https://mc.manuscriptcentral.com/e-videos .
